# Comparative bibliometric trends of microplastics and perfluoroalkyl and polyfluoroalkyl substances: how these hot environmental remediation research topics developed over time †

**DOI:** 10.1039/d1ra09344d

**Published:** 2022-02-09

**Authors:** Reza Bakhshoodeh, Rafael M. Santos

**Affiliations:** Department of Civil, Environmental and Mining Engineering, University of Western Australia Perth Australia; School of Engineering, University of Guelph Guelph Ontario Canada santosr@uoguelph.ca

## Abstract

Microplastics (MPs) and per- and polyfluoroalkyl substances (PFAS) are ubiquitous in the environment due to consumer and industrial use. These compounds are very persistent in the environment and human body, which has made them hot environmental topics in recent years; but how this did come about? What factors have been driving the trends of their publication records, the public concern over environmental and public health issues caused by these pollutants, and the collaboration between scientists, regulators, and policymakers to solve these problems? In this paper, to understand these factors and contrast them between the two hot topics (“PFAS” and “MPs”), the changes in the bibliometric and scientometric trends of their publication records (extracted from the Web of Science from 1990 to 2020) have been visualized over time based on different classification perspectives, such as year, country, source, and organization. According to the analyses performed on these records, utilizing publication ratios and principal component and cluster analyses, in recent years (beginning in 2018) research topics related to MPs have surpassed PFAS topics. In addition, the economic, social, and geographical conditions of the top 20 countries with the highest number of publications in MPs and PFAS were explored to identify which countries are most concerned about each of these topics and why. For instance, PFAS research topics were more prevalant in countries with larger water areas compared to land area; while MPs topic were more prevelant in countries that produced more plastic wastes, and had higher landfilling and recycling rates and greater proportion of treated wastewater.

## Abstract 2

Bibliometrics point to the hottest areas of science and policy working to reverse the environmental impacts of PFAS and microplastics.

## Graphical abstract

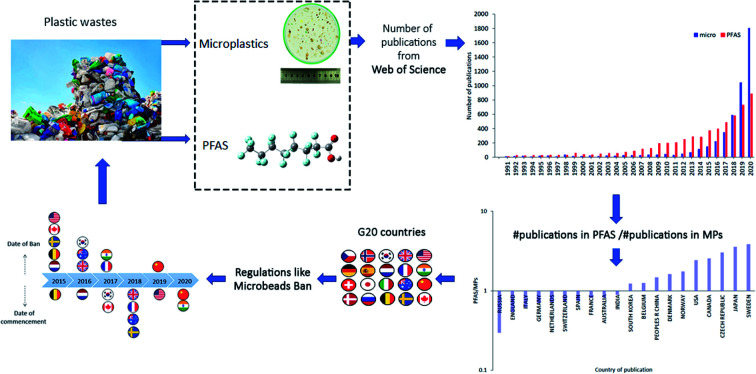

## Introduction

Plastics have played an important role in modern life, as they are used in many everyday products due to their unique properties such as low cost, lightweight, and chemical stability ^1^. Over a 13 year period (2004–2018), global plastics production increased steadily, rising from 224 to 360 million tonnes^2^.

While plastics have been used in medical, food packaging, technological devices, and other applications, their resistance to degradation makes them a serious environmental problem that endangers human and animal health.^3^ According to the available reports, only about 6% of these plastics have been recycled, 39% have been incinerated, and 31% have been landfilled and the remaining has ended up in the environment and oceans;^4^ where they disintegrate and break up into very small solid particles, called perfluoroalkyl and polyfluoroalkyl substances (PFAS) and microplastics (MPs). It has been estimated that, by 2050, more than 12 000 megatons of plastic will have ended up in the environment and landfills.^5^

PFAS and MPs are a class of highly environmentally stable anthropogenic environmental pollutants that are commonly found in the aquatic environment, wildlife, and humans. They have been used in a variety of industries worldwide, including the production of water repellent clothing, water and stain proofing agents, paints, lubricants, cookware, and fire-fighting foams.

PFAS are a group of man-made unique chemically stable compounds that includes perfluorooctanoic acid (PFOA), perfluorooctanesulfonic acid (PFOS), GenX, and many other chemicals. GenX is a trade name for a technology that uses no PFOA to produce high-performance fluoropolymers such as nonstick coatings. PFAS can be released into the environment, including the air, soil, and water. However, this property also makes them recalcitrant and persistent in nature, earning them the moniker “forever” chemicals.^6^

Microplastics, which are defined as plastic polymer particles smaller than 5 mm in size^7^ and are classified as primary and secondary MPs, are another serious environmental pollution.^8^ Primary microplastics are commonly found in manufacturing and packaging and cosmetic products^9^ and secondary microplastics will be formed as a result of degradation *via* physical or chemical processes when larger pieces are fragmented/broken down to less than 5 mm.^9^ According to some studies, by 2100, it has been estimated that about 2.5 × 10^7^ to 1.3 × 10^8^ tons of microplastics will be floated in the ocean.^10^

Chemicals and pollutants are both persistent in the environment and in the human body, which means they do not degrade and can accumulate over time (*i.e.*, the chemicals bioaccumulation). They can be easily found commercial household products, non-stick cookware (*e.g.*, Teflon), and fire-fighting foams; workplace, including production facilities or industries such as electronics manufacturing; drinking water; and in living organisms, including fish, animals, and humans, where these pollutants have the ability to build up and persist over time.

It is also critical to emphasise that one type of PFAS, polymeric PFAS, can degrade into microplastics, which highlights a connection between PFAS and MPs.^11^ Fluoropolymers, side-chain fluorinated polymers, and poly- or perfluoropolyethers are all members of this polymeric PFAS group.^12,13^ PTFE (polytetrafluoroethylene) and PVF (polyvinylidene fluoride) can be found in the environment as secondary microplastics or as primary microplastics that are purposefully created. In addition to polymeric PFAS and PFAS-coated plastics and textiles, PFAS can adsorb onto microplastics in the environment and possibly desorb in aquatic species but not in the human gut.^14,15^ Therefore, microplastics and PFAS are frequently found in the environment together, and recent research suggests that microplastics may increase PFAS toxicity.^16^ In addition, because of the specific environmental and health-related outcomes associated with PFAS and MPs, such as harmful impact on the environment, human health, and toxicological risks for living organisms, these two topics are considered hot topics in the manufacturing and environmental arenas.

In the last years, many journals, conferences, funding organization, and scholarly publications have spurred investigations of these topics at rates not seen before. The goal of this article is thus to compare the bibliometric and scientometric data on MPs and PFAS from 1990 to 2020 in order determine their trends over time. Because traditional review papers^17–22^ are incapable of revealing trends and relationships on a specific topic, bibliometric analysis has been introduced for this purpose.

Bibliometric analysis is a popular technique that has recently been used to investigate the internal relationships in a body of scientific outputs published in the literature. This method is useful for researchers who are interested in but unfamiliar with a specific field in order to quickly understand the status of that field. Several bibliometric studies have investigated various topics related to these pollutants and attempted to visualise the development of PFAS and MPs topics.^23–28^ These studies frequently make use of the most comprehensive literature databases, such as Web of Science and Scopus. Such studies are also common to span several decades and cover topics ranging from regional to global in scope.

The search string used to retrieve publications from databases is an important aspect of bibliometric studies. Authors frequently use keyword combinations and variations because using too restrictive or specific keywords (*e.g.*, simply “microplastics”) can result in an incomplete search record. For example, Zhang *et al.*^28^ utilized [“microplastic” OR “microplastics” OR “micro-plastic” OR “micro-plastics” OR “micro-sized plastic” OR “micro-sized plastics”]; Pauna *et al.*^27^ utilized [“microplastic* AND “marine”]; Sorensen *et al.*^29^ utilized [((microplastic* OR nanoplastic* OR “plastic particle*” OR microbead* OR microfibre* OR microfiber*) AND (marine OR litter OR pollution OR toxic* OR environment* OR health* OR ingestion OR debris OR waste OR sediment*))]; Podder *et al.*^23^ utilized [“pfas”, “perfluorinated” OR “polyfluorinated” AND “sources” AND “occurrences” AND “fate AND transport” AND “remediation”]. The search in the study of Zhang *et al.*^28^ was more general and comprehensive than others; they covered the geographical distribution and published sources of MPs research and elaborated the hotspots in this field. On the other hand, Pauna *et al.*^27^ studied specifically on marine pollutions and microplastics and tried to find the current hot aspects and knowledge gaps in this area.

Apart from the novel comparative between MPs and PFAS topics, another distinguishing feature of this article is its use of publication ratio values to compare these two topics. The publication ratios were calculated by dividing the number of publications in a category in one record by the number of publications in another record, allowing us to distinguish and contrast MPs research from PFAS research. Santos and Bakhshoodeh (2021) pioneered this method by comparing trends in climate change/global warming/climate emergency research to general climate research^30^.

The current study has a global scope and spans 30 years of data aimed to highlight key moments in the publication record and scientific advancement histories, as well as important variables affecting the trends by answering the following research questions: (i) what are the dynamics of the conceptual structure of PFAS *versus* MPs research; (ii) when did the scientific record in PFAS *versus* MPs research become more enriched; (iii) in which countries have these pollutants have become the dominant topic, and are there any relationships between such countries and the dominant scientific topic?

## Methodology

**Fig. 1:**
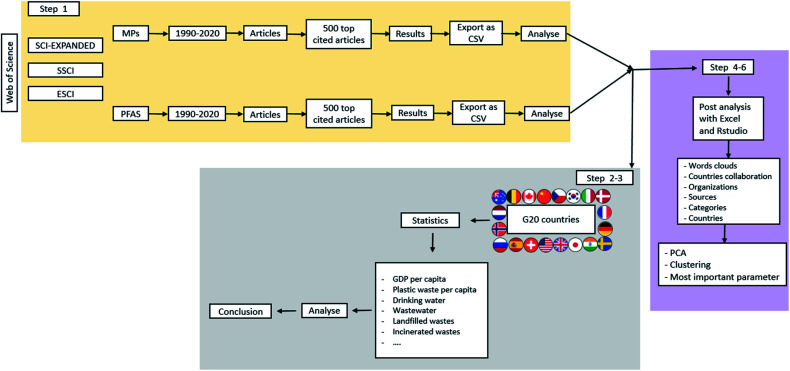
Protocol used for the bibliometric study.

The scientific literature was searched using Web of Science (WoS) and the relevant publication data was collected for analysis. On June 15th, 2021, the searches were executed (from 1990 to complete 2020 data); all data were collected in a short period of time to obtain a snapshot of the publication record. This bibliometric study's protocol (Fig. 1) was similar to another study on comparative bibliometric trends of publications in two different topics (climate and climate change);^30^ this protocol is divided into five steps, which are detailed below.

### Step one

The time span of 1990 to 2020 was used for the search and all indexes within the Web of Science Core Collection, namely: Science Citation Index Expanded: SCI-EXPANDED (1900–2020), Emerging Sources Citation Index: ESCI (2015–2020), and Social Sciences Citation Index: SSCI (1900–2020). The two search strings used were: (i) TOPIC: ((“microplastic*” OR “micro-plastic*” OR “nanoplastic” OR “nano-plastic*”)); and (ii) TOPIC: ((((“pfas” OR ”*fluoroalkyl” OR “per* fluor*” OR “poly* fluor*”) NOT (“polyvinylidene fluoride” OR “polyvinylidene difluoride” OR “PVDF”)) AND (“enviro*” OR “fate” OR “pollut*” OR “remediat*” OR “toxic*” OR “soil” OR “food” OR “contamina*” OR ”*water” OR “waste*”))). The former search string was used to collect papers on microplastics (MPs) research, while the latter search string was used to collect papers on perfluoroalkyl and polyfluoroalkyl substances (PFASs).

### Step two

The downloaded database was sorted based on the number of publications in MPs and PFAS records. The document type was then refined to ‘'Article,' and these searches yielded 4 990 papers on the MPs topic and 5 797 papers on the PFAS topic. At the next step, the top 20 countries (G20) having the highest number of publications in MPs and PFAS were selected for further analysis. In addition, the number of publications was normalised based on the total number of publications in each country in order to normalise publication data and reduce the impact of each country's population.

### Step three

The statistical data of the G20 countries were downloaded to answer the research questions, including GDP per capita, plastic waste production per capita, manufacturing rate, drinking water contribution, *etc.*

### Step four

The search results were at first analysed using the “Analyse Results” feature of WoS. The analysis page allows one to download tab-delimited text files containing a set of publication data based on the WoS category chosen. Data files were obtained for the four categories listed below: publication years, source titles, organizations-enhanced, and countries/regions. The text file data was then imported into Microsoft Excel for further processing and analysis. These data and analyses allowed us to answer the research questions.

### Step five

A bibliometric analysis, which included keyword co-occurrence, country collaboration, affiliations, and most relevant words, was also performed on the full search results, which were exported from WoS as a bibtex or CSV file, using the bibliometrix package in RStudio software (Version 1.2.5001©2009–2019 RStudio, Inc).^31^ The bibliometrix R-package (http://www.bibliometrix.org) is a collection of tools for quantitative research in bibliometrics and scientometrics. It is written in R, an open-source environment and ecosystem.^32^ This package also calculates and ranks country production, journal sources, and country collaborations using meta-data from Web of Science citations.

### Step six

Principal Component Analysis (PCA), a multivariate statistical method, was used as a data descriptive tool to uncover patterns and determine which factor has the greatest impact on the publication ratio, as well as to reduce the dimensionality of the downloaded dataset (steps 1–3); this technique can reduce the dimensionality of the original variables by computing the principal components, which are a new set of variables that represents a linear combination of the original variables.^33,34^ This method was adapted from Fantucci *et al.*^35^ and helps to gain a better understanding of the most important factors affecting the number of publications in different countries by using PCA analysis. As a descriptive tool, PCA does not require any distributional assumptions; however, a normal distribution should be assumed for inferential purposes. The analyses were carried out with the help of the RStudio software and prcomp function,^36^ and the data were standardised (mean = 0 and standard deviation = 1) by using the centre = TRUE and scale = TRUE parameters.^33,34^

In addition to the PCA, clustering analysis was performed to discover potentially significant group of countries that are focusing more on a specific research area. The analyses were carried out with the help of the RStudio software and the factoextra and cluster functions.^36,37^ Two clusters were defined using *K*-means clustering, which was used for partitioning a given data set into a set of k groups (*i.e.*, k clusters).

## Results and discussion

**Fig. 2:**
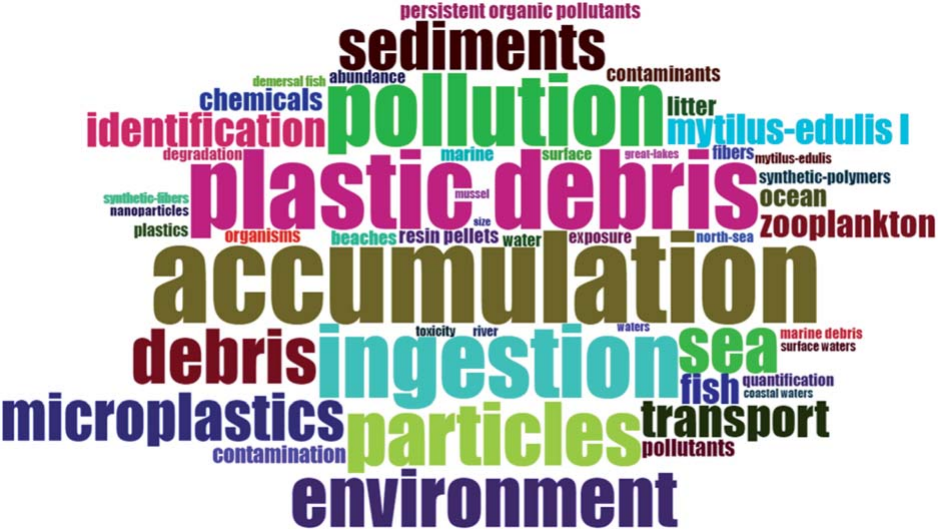
Word cloud of keywords from top 100 most cited papers on MPs research.

**Fig. 3:**
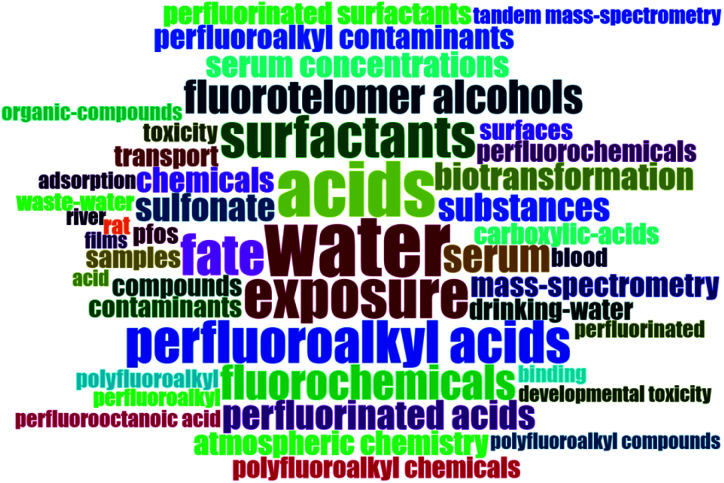
Word cloud of keywords from top 100 most cited papers on PFAS research.

The word clouds generated for the keywords extracted from the top 100 most cited papers in each record are shown in Fig. 2 and 3. Table 1 also shows the top ten words used in the top 100 cited papers in each record. Pollution, environment, water, and contaminants are some of the main words on both clouds. The conclusion from word clouds helps us study in-depth the impact of different parameters to answer the research questions like the impact of drinking water, surface water, groundwater, wastewater, marine environment, and fish on the publications rate on MPs and PFAS.

**Table 1:** Word frequency of top ten used words in Fig. 2 and 3

MPs record		PFAS record	
Word	Frequency	Word	Frequency
Marine-environment	209	Perfluorooctane sulfonate	83
Accumulation	164	Water	62
Plastic debris	141	Perfluorinated compounds	60
Ingestion	130	Acids	55
Pollution	128	Exposure	46
Particles	111	Perfluorooctane sulfonate pfos	45
Environment	103	Perfluoroalkyl acids	42
Debris	98	Surfactants	42
Sea	95	Fate	41
Sediments	86	Fluorochemicals	34

According to the findings in Table 1, and also the importance and relationship of these pollutants and plastic production with economic, social, and geographic factors such as GDP per capita, plastic waste production, freshwater availability, fish and meat consumption, *etc.*, the relationship between these parameters and the number of publications in each topic and the publication ratio will be investigated separately. The subsequent seven sub-sections are organized into the following categories of data collection and analysis of the publication records: (i) Year of publication; (ii) Source of publication (areas of interest and institutions/universities and organizations); (iii) Country (corresponding author's) of publication; (iv) Economic factors; (v) Landfilled and incinerated wastes; (vi) Drinking water; and (vii) Wastewater. This is followed by a sub-section on PCA and clustering analyses and a sub-section on the topic of microbeads and its regulation.

### Year of publication

**Fig. 4:**
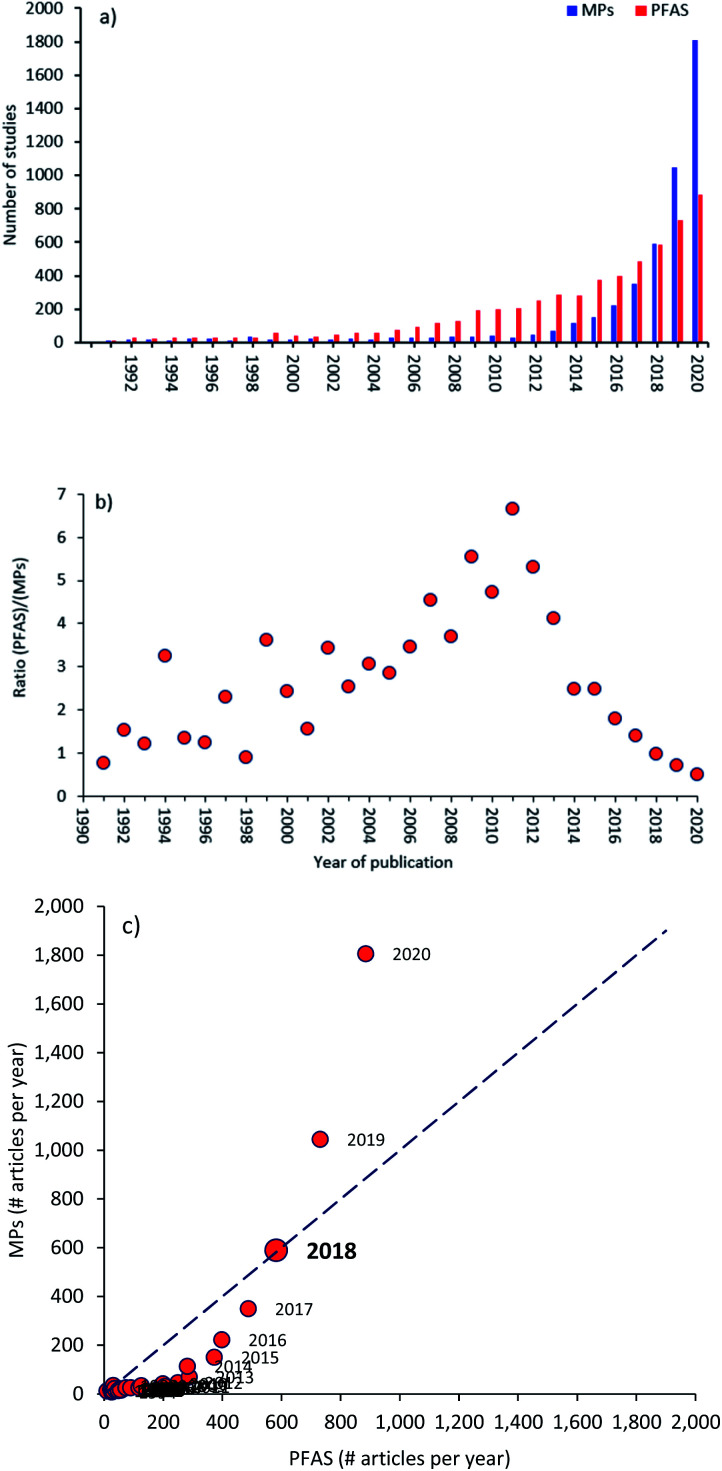
(a) The annual number of publications on PFAS and MPs research topics from 1990 to 2020; (b) the publication ratio (PFAS/MPs) as a function of time (years); (c) the number of publications per year in the MPs record *versus* those in the PFAS record (dashed line illustrates the 1 : 1 mark).

The data analysis for each year of publication, from 1990 to 2020, is shown in Fig. 4. WoS was used to compile the number of articles published in the two publication records (MPs and PFAS) each year (Fig. 4a). For each year, a ratio of the number of articles in the MPs record to the number of articles in the PFAS record was calculated. This ratio is plotted as a function of time in Fig. 4b and c to visualize when the scientific record became more enriched in PFAS *versus* MPs research; that is when the ratio surpasses a value of one. This happened in 2018, and the ratio has since risen to 2.04 in 2020 (an all-time high); the exceptions in 1991 and 1998 were due to a low number of PFAS publications. Since 2000, the ratio has nearly doubled every year (in fact, it increases 15 out of 21 times, and every year since 2011).

The number of publications in both records has increased by orders of magnitude in recent years, as illustrated in Fig. 4c. In 2007, the PFAS record exceeded 100 publications in a single year, whereas the MPs record did not reach that milestone until 2014. The MPs record also exceeded 1 000 publications in 2019, whereas the PFAS record indicated that the number of studies in this field had not exceeded 1 000 in a year. This corresponds almost exactly with the 2011 threshold, when the MPs record surpassed the PFAS in terms of number of publications per year (1 045 for MPs and 731 for PFAS, respectively) (Fig. 4a). Fig. 4c shows the 2018 records above the dashed line, and the pre-2018 records below the dashed line.

After the Stockholm Convention on Persistent Organic Pollutants in 2001, the publication rate of PFAS topics increased; also, the publications ratio of PFAS/MPs experienced a rising trend from 2001 to 2011. Then, since 2011, due to the Fifth International Marine Debris Conference (5IMDC) and first United Nations Environment Assembly developed by the United Nations Environment Programme (UNEP) with the focus of an international framework to reduce marine plastic pollution, the number of MPs publications has significantly increased, which has resulted in increasing the MPs/PFAS ratio. Fig. 4b shows the annual number of publications on both topics between 1990–2020. In addition, Sweden as the host of the Stockholm Convention on Persistent Organic Pollutants has the highest contribution on PFAS topics.

### Source of publication

#### Areas of interest of the published research

Table 2 presents the most prevalent areas of interest among the published research in MPs and PFAS. For MPs, environmental science was at the top (3275; 61.3%), followed by material science (650; 12.2%) and environmental engineering (517; 9.7%), while for PFAS, the order was environmental science (3069; 37.3%), chemistry (1857; 22.5%), and material science (1096; 13.3%).

**Table 2:** Ranking of areas of interest of the published research in the field of MPs and PFAS within the period of 1990–2020

SCR^a^ (↓)	Areas of interest	Number of MPs publications (%) (↓)	Number of PFAS publications (%)
1^st^	Environmental sciences	3275 (61.3)	3069 (37.3)
2^nd^	Materials science	650 (12.2)	1096 (13.3)
3^rd^	Environmental engineering	517 (9.7)	804 (9.8)
4^th^	Chemistry	336 (9.3)	1857 (22.5)
5^th^	Pharmacology, toxicology, and pharmaceutics	290 (5.4)	755 (9.2)
6^th^	Chemical engineering	113 (2.1)	360 (4.4)
7^th^	Physics	108 (2)	158 (1.9)
8^th^	Agricultural and biological sciences	53 (1)	119 (1.4)

a SCR: standard competition ranking.

Table 3 presents the top journal ranking for MPs and PFAS publications. *Journal of Environmental Pollution* was at the top with 474 articles (27%), followed by *Journal of Science of The Total Environment*, which had 419 articles (24%), and *Environmental Science & Technology* with 219 articles (13%). On the other hand, for PFAS, the order was *Environmental Science & Technology* (440; 18%), *Chemosphere* (341; 14%), and *Science of The Total Environment* (271; 11%). All of the journals that have been listed in the list of top 20 journals had impact factors greater than 2 with the average impact factor (IF) of 6.7 (45% of the journals with IF more than 7; 20% of the journals with IF between 5–7; 35% of the journals with IF between 2–5). The average IF of the top three journals mentioned earlier was 9.53 and 9.20 for MPs and PFAS, respectively. In addition, about 35% of articles in MPs were published in the *Environmental Pollution* and in *Environmental Science and Pollution Research*, titles which have the word “pollution”, while in PFAS, about 30% of articles were published in *Science of The Total Environment* and in *Environmental Science & Technology*, which are more about environmental science.

**Table 3:** Ranking of the top 20 journals in which MPs and PFAS related articles were published

SCR^a^^,^^b^ (↓)	Journal	Number of MPs publications (%) (↓)	Number of PFAS publications (%)	IF^c^	MPs/PFAS
1^st^	*Environmental Pollution*	474 (27)	227 (9)	8.07	2.09
2^nd^	*Science of The Total Environment*	419 (24)	271 (11)	7.96	1.55
3^rd^	*Environmental & Science Technology*	219 (13)	440 (18)	9.03	0.50
4^th^	*Chemosphere*	155 (9)	341 (14)	7.09	0.45
5^th^	*Environmental Science and Pollution Research*	118 (7)	90 (4)	4.22	1.31
6^th^	*Journal of Hazardous Materials*	89 (5)	68 (3)	10.59	1.31
6^th^	*Water Research*	89 (5)	74 (3)	11.24	1.20
7^th^	*Ecotoxicology and Environmental Safety*	50 (3)	50 (2)	6.29	1.00
8^th^	*Environmental Toxicology and Chemistry*	33 (2)	96 (4)	3.74	0.34
8^th^	*Environment International*	33 (2)	169 (7)	9.62	0.20
9^th^	*Environmental Research*	27 (2)	104 (4)	6.50	0.26
10^th^	*Environmental Science Technology Letters*	12 (1)	52 (2)	7.65	0.23
11^th^	*Journal of Applied Polymer Science*	6 (<1)	46 (2)	3.12	0.13
12^th^	*RSC Advances*	4 (<1)	47 (2)	3.36	0.09
13^th^	*Langmuir*	3 (<1)	109 (4)	3.88	0.03
13^th^	*Journal of Chromatography*	3 (<1)	72 (3)	4.76	0.04
14^th^	*Macromolecules*	2 (<1)	51 (2)	5.98	0.04
15^th^	*Environmental Health Perspectives*	0 (0)	63 (3)	9.03	0.00
15^th^	*Applied Surface Science*	0 (0)	41 (2)	6.71	0.00
15^th^	*Journal of Fluorine Chemistry*	0 (0)	83 (3)	2.05	0.00

a SCR: standard competition ranking.

b Ranked based on the number of publications; equal journals have the same ranking number, and then a gap is left in the ranking numbers in the MPs topics.

c IF: impact factor; reported according to Journal Citation Reports (JCR) 2020.

#### Institutions/universities and organisations

Table 4 shows the top 20 most productive institutions/universities and organisations, ordered by ascending MPs/PFAS ratio. The Helmholtz Association in Germany was the most prolific institution for the number of publications in MPs, with 124 articles (13%) and MPs/PFAS of 31, followed by the University of Chinese Academy of Sciences in China, with 107 articles (11.3%) and MPs/PFAS of 11.9. China had the most institutions/universities on the list of the top 20 most prolific institutions/universities, with six institutions, followed by England, Germany, France, and Australia, each with two institutions.

**Table 4:** Ranking of the top 20 most highly productive institutions/universities in MPs research between 1990–2020

Name of the institution (↓)	Country	Number of MPs publications (%)	Number of PFAS publications (%)	MPs/PFAS	2020 rank^a^
Aalborg University	Denmark	23 (2.4)	5 (1.4)	4.6	326
CNRS Institute of Ecology Environment INEE	France	30 (3.1)	8 (2.3)	3.7	na
Commonwealth Scientific Industrial Research Organisation CSIRO	Australia	24 (2.5)	6 (1.7)	4	241^b^
Environment Climate Change Canada	Canada	24 (2.5)	26 (7.4)	0.9	na
Helmholtz Association	Germany	124 (13)	4 (1.1)	31	na
Italian Institute for Environmental Protection Research ISPRA	Italy	35 (3.7)	6 (1.7)	5.8	na
Nanjing University	China	47 (4.9)	42 (12)	1.1	131
Qingdao Natl Lab Marine Sci Technol	China	35 (3.7)	38 (10.8)	0.9	na
Research Center for Eco Environmental Sciences RCEES	China	20 (2.1)	15 (4.3)	1.3	na
Russian Academy of Sciences	Russia	150 (15.7)	50 (14.2)	3	7^b^
RWTH Aachen University	Germany	25 (2.6)	17 (4.8)	1.5	165
Universite De Bretagne Occidentale	France	22 (2.3)	20 (5.7)	1.1	631^b^
University of California System	USA	75 (7.9)	14 (4)	5.3	na
University of Chinese Academy of Sciences CAS	China	107 (11.2)	9 (2.6)	11.9	27^b^
University of Gothenburg	Sweden	26 (2.7)	21 (6)	1.2	180
University of London	England	27 (2.8)	5 (1.4)	5.4	na^c^
University of Plymouth	England	75 (7.9)	26 (7.4)	2.9	601-650
University of Queensland	Australia	27 (2.8)	20 (5.7)	1.3	47
Yantai Institute of Coastal Zone Research CAS	China	31 (3.2)	7 (2)	4.4	562^b^
Zhejiang University	China	26 (2.7)	12 (3.4)	2.2	45

a According to QS World University Rankings 2022.

b According the SCIMAGO Institutions Rankings.

c University of London is ambiguously used for University College London (8), Queen Mary University of London (117), and other institutions.

Table 5 shows the list of the top 20 prolific institutions/universities and organizations sorted by descending MPs/PFAS ratio. The Communaute Universite Grenoble Alpes in France was the most prolific institution in terms of PFAS publications, with 395 articles (16.5%), followed by Shihezi University in China with 156 articles (6.5%). The countries with the most institutions/universities in the top 20 most prolific institutions/universities were the United States, China, and France, with 5, 3, and 2 institutions/universities, respectively.

**Table 5:** Ranking of the top 20 most highly productive institutions/universities in PFAS research between 1990–2020

Name of the institution (↓)	Country	Number of MPs publications (%)	Number of PFAS publications (%)	MPs/PFAS	2020 ranking^a^
Aarhus University	Denmark	15 (22.5)	79 (3.3)	0.19	155
China Agricultural University	China	16 (24)	74 (3.1)	0.22	601–650
China University of Mining Technology	China	4 (6)	142 (5.9)	0.03	800–1000
Communaute Universite Grenoble Alpes	France	6 (9)	395 (16.4)	0.02	314
FSC Millport	England	2 (3)	143 (5.9)	0.01	na
Inst Antartico Chileno	Chile	1 (1.5)	80 (3.3)	0.01	na
Inst Mongolovedeniya Buddol Tibetol Sb Ras	Russia	1 (1.5)	113 (4.7)	0.01	na
Nha Trang Univ	Vietnam	1 (1.5)	97 (4)	0.01	na
Norwegian Institute for Air Research	Norway	3 (4.5)	91 (3.8)	0.03	695^b^
Pyhajarvi Inst	Finland	1 (1.5)	79 (3.3)	0.01	na
Rhode Isl Sch Design	USA	1 (1.5)	70 (2.9)	0.01	na
Shihezi University	China	1 (1.5)	156 (6.5)	0.01	676^b^
Umweltbundesamt UBA	Germany	1 (1.5)	96 (4)	0.01	na
United States Air Force	USA	3 (4.5)	129 (5.4)	0.02	na
Univ Favaloro	Argentina	1 (1.5)	73 (3)	0.01	na
Universite De Bourgogne	France	1 (1.5)	126 (5.2)	0.01	501–600
University of Mississippi	USA	6 (9)	130 (5.4)	0.05	801–1000
University of Nairobi	Kenya	1 (1.5)	144 (6)	0.01	1001–1200
University of Wyoming	USA	1 (1.5)	70 (2.9)	0.01	801–1000
World Ocean	USA	1 (1.5)	114 (4.7)	0.01	na

a According to QS World University Rankings 2022.

b According the SCIMAGO Institutions Rankings.

According to Tables 4 and 5, the average MPs/PFAS of universities and institutions were 3.65 and 6.35, respectively. This suggests that academic organisations may conduct more PFAS research, whereas governmental organisations and institutions may be more focused on MPs research.

### Country of publication

**Fig. 5:**
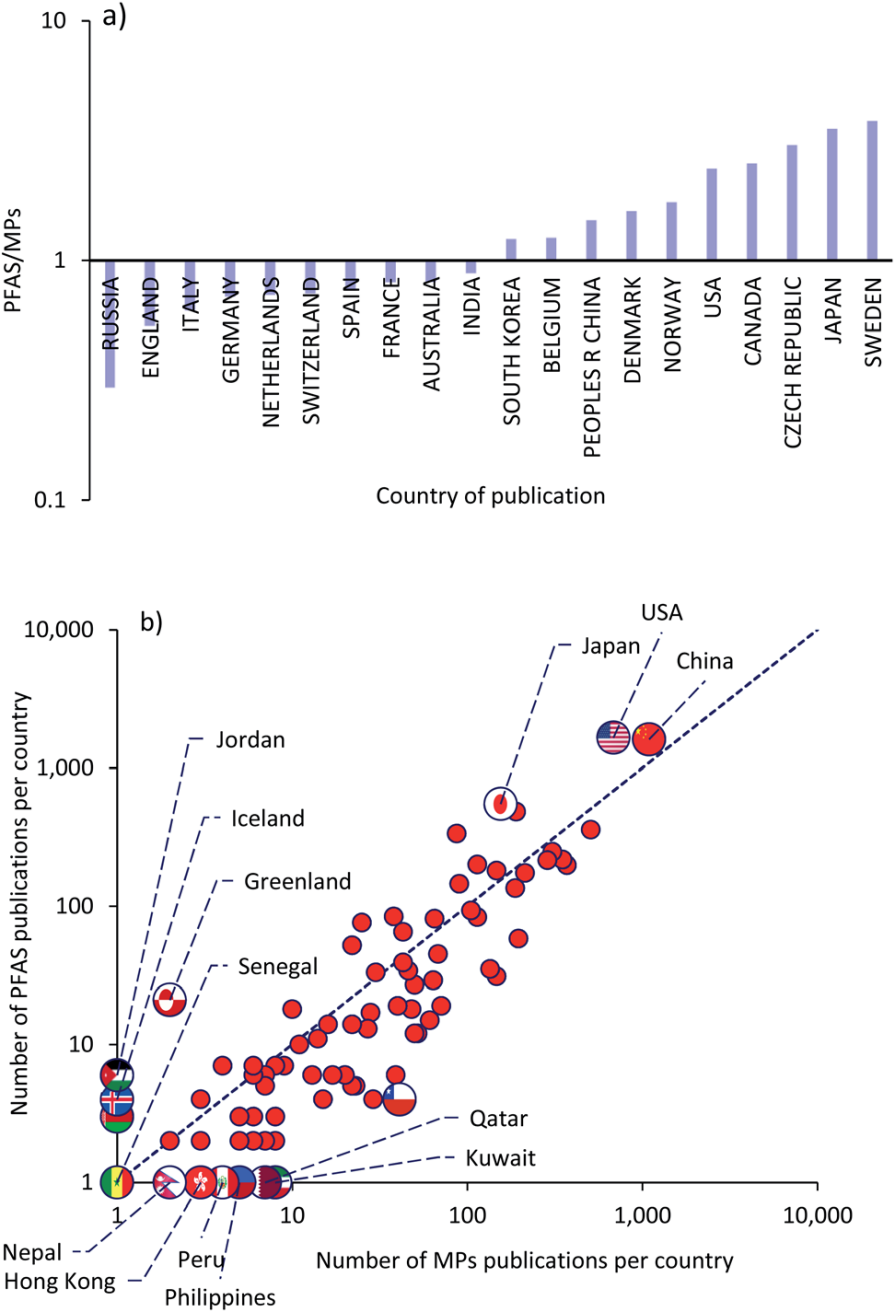
Data for MPs and PFAS records for country/region of publication: (a) publication ratio (PFAS/MPs) of G20 countries, ordered from lowest to highest ratio (axis labels are provided for a selection of states); (b) number of publications per country in the MPs record *versus* those in the PFAS record (dashed line illustrates the 1 : 1 mark) for all countries.

Fig. 5a presents the data analysis for the country of publications for the top 20 countries (G20) with the highest publication records from 1990 to 2020. A ratio of the number of articles in the MPs record to the number of articles in the PFAS record was calculated for each country. In Fig. 5a, this ratio was plotted for each country in descending order from largest to smallest. Countries with a ratio greater than one have been more involved in MPs research, while those with a ratio less than one have been more involved in PFAS research. The total land and water areas of countries with a ratio greater than one were equivalent to 19.7 and 24.9% of the total world's, respectively, while for countries with a ratio less than one, these ratios were 19.3 and 35.5%. According to these ratios, PFAS topics were more prevalent in countries with larger water areas compared to land area, whereas the trend for MPs was inverse (Tables 6 and 7).

**Table 6:** The number of publications in MPs, PFAS (normalised as a fraction of the total number of articles in each country, times 10 000 for legibility), and the ratio of MPs/PFAS for the G20 countries with the information of plastic wastes production and management

Country	#MPs (× 10 000)	#PFAS (× 10 000)	MPs/PFAS (↓)	GDP per capita ($US)^39^	Percent of global manufacturing (%)^38^	Plastic waste per capita (kg per capita)^40^	Incineration rate (%)^41^	Recycle rate (%)^41^	Landfill rate (%)^41^
Russia	21.71	6.42	3.38	11 585	—	0.11	—	—	—
England	15.12	8.07	1.87	42 354	1.80	0.22	23	41	34
Italy	23.85	14.79	1.61	33 566	2.30	0.13	21	41	38
Germany	21.56	15.23	1.42	46 467	5.80	0.49	34	66	—
Netherlands	22.58	16.22	1.39	52 229	1.00	0.42	48	58	1
Switzerland	17.30	12.59	1.37	85 300	1.00	—	49	51	0.0
Spain	25.64	19.21	1.33	29 564	2.00	0.28	10	30	60
France	13.86	11.24	1.23	40 380	1.90	0.19	33	28	28
Australia	19.53	15.88	1.23	55 057	0.00	0.11	1	41	58
India	7.80	6.91	1.13	2100	3.00	0.01	—	—	—
South Korea	15.94	19.63	0.81	31 846	3.30	0.11	24	59	16
Belgium	14.02	17.48	0.80	46 414	4.00	0.08	43	55	1
Peoples R China	26.31	38.80	0.68	10 216	28.40	0.12	—	—	—
Denmark	23.41	37.71	0.62	60 213	—	0.05	54	44	2
Norway	43.36	76.07	0.57	75 826	—	0.28	57	39	—
USA	7.18	17.38	0.41	65 279	16.60	0.34	3	12	54
Canada	11.90	30.31	0.39	46 326	1.00	0.09	4	24	72
Czech Republic	10.50	31.94	0.33	23 494	—	—	—	—	—
Japan	5.97	21.21	0.28	40 113	7.20	0.17	71	25	1
Sweden	13.29	51.17	0.26	51 686	—	0.05	50	50	0.0

**Table 7:** The number of publications in MPs, PFAS (normalised as a fraction of the total number of articles in each country, times 10 000 for legibility), and the ratio of MPs/PFAS for the G20 countries with the information of freshwater and drinking water

Country	#MPs (× 10 000)	#PFAS (× 10 000)	MPs/PFAS (↓)	% of world land area^52^	% of world water area^52^	Fresh water availability (billion m^3^)^53^	Fresh water availability (m^3^ per capita)^53^	Drinking water from groundwater (%)^54^	Drinking water from surface water (%)^54^
Russia	21.71	6.42	3.38	11.00	4.20	—	29 842	8.60	—
England	15.12	8.07	1.87	0.20	0.69	145	2195	20.00	80.00
Italy	23.85	14.79	1.61	0.20	2.40	183	3015	28.00	72.00
Germany	21.56	15.23	1.42	0.20	2.30	107	1295	70.00	30.00
Netherlands	22.58	16.22	1.39	0.03	18.4	11	642	60.00	40.00
Switzerland	17.30	12.59	1.37	0.03	3.10	40	4780	40.00	60.00
Spain	25.64	19.21	1.33	0.30	1.30	111	2387	19.00	75.00
France	13.86	11.24	1.23	0.40	0.52	—	2989	62.00	28.00
Australia	19.53	15.88	1.23	5.20	0.76	492	19 998	0.50	—
India	7.80	6.91	1.13	2.00	9.60	1446	1080	68.00	32.00
South Korea	15.94	19.63	0.81	0.10	0.30	65	1263	33.10	70.00
Belgium	14.02	17.48	0.80	0.00	0.82	12	1055	—	—
Peoples R China	26.31	38.80	0.68	6.30	2.80	2813	2029	18.00	82.00
Denmark	23.41	37.71	0.62	0.00	0.00	6	1041	—	—
Norway	43.36	76.07	0.57	0.20	6.00	382	72 390	—	10.00
USA	7.18	17.38	0.41	6.10	4.00	2818	8668	15.00	85.00
Canada	11.90	30.31	0.39	6.10	8.90	2850	77 985	0.30	99.00
Czech Republic	10.50	31.94	0.33	0.05	2.12	13	1249	—	—
Japan	5.97	21.21	0.28	0.20	3.60	430	3392	23.00	77.00
Sweden	13.29	51.17	0.26	0.30	8.90	171	17 002	50.00	50.00

Fig. 5b shows a different perspective on the country publication records. It is possible to see a focusing effect about the 1 : 1 dashed line by plotting the number of MPs publications for each country *versus* the number of PFAS publications in the same country. Countries with a higher number of publications are more likely to be involved in recent research and thus have more MPs articles than PFAS articles.

To answer the question, “in which countries (G20) have these pollutions become the dominant topic and are there any relationships between countries and the dominant scientific topic?”, the economic, social, and geographical situations were discussed in depth. Some of these factors include GDP per capita, plastic and plastic waste production per capita, the amount of landfilled, recycled, and incinerated waste, the percentage of global manufacturing rate, fish, meat, and egg consumption per capita, and fresh water availability. The most important parameters will be discussed in more detail in following section.

### Economic factors

Over the last few decades, technological advancements, changing lifestyles, commercial and economic growth have resulted in increased waste generation in many countries, which has a direct relationship with GDP per capita. Table 6, Fig. S1 and S2† in the ESI show the GDP per capita and percent of global manufacturing of G20 countries according to data published by the United Nations Statistics Division.^38^

According to the findings, there was no significant relationship between GDP per capita and the number of publications in different countries; 50% of the countries had a ratio greater than one and 50% had a ratio less than one (Fig. S1†). For example, Norway and Switzerland almost had the same GDP per capita, but Norway's research focused more on PFAS while for Switzerland was on MPs. To understand the trends of these topics on publication rates, we must investigate and cover the impact of various other factors such as drinking water, wastewater, waste managements, *etc.*

The relationship between percent of global manufacturing and the publication rates of G20 countries has been shown in Fig. S2† and Table 6. The findings revealed a negative relationship between the ratio of MPs/PFAS and manufacturing rates (the Pearson coefficients of −0.4), implying that increasing a country's manufacturing rate decreased the ratio, which could be a decrease in the number of MPs publications or an increase in PFAS publications. Also, the mean manufacturing rate for the countries with the MPs/PFAS ratios lower than one was 10.1 while for ratios greater than one was 2.1.

Furthermore, the Pearson correlation between per capita plastic waste production and the ratio of MPs/PFAS and MPs publications revealed a positive correlation (0.2), indicating that the number of MPs publications was higher in countries with higher per capita plastic waste production than in countries with lower per capita plastic waste production (Fig. S3 in the ESI†).

### Wastes: landfilled and incinerated

Waste is another important economic and social factor that can contain and carry significant amounts of MPs and PFAS. According to reported statistics over the last 50 years, more than 5800 million megatons of plastics produced; 4900, 800, and 100 million megatons were landfilled, incinerated, and recycled, respectively.^42^

Physical changes and biochemical reactions in landfills have been regarded as dangerous processes, transforming plastic wastes into severe environmental problems such as microplastics resulting from plastic fragmentation, which can be discharged and spread from landfills to surrounding environments *via* leachate and air.^43–45^ Landfills are one of the potential point sources for PFASs and MPs in groundwater.^42^ Fig. S4 in the ESI† shows that in countries that landfills account for more than 20% of waste disposal, MP/PFAS levels were higher than in countries where landfills were not the primary waste treatment method, with the exception of Canada and the United States, which account for about 13% of the total world landmass. Furthermore, the Pearson coefficients showed a positive correlation (the Pearson coefficients of 0.4) between the ratio of MPs/PFAS and the amount of landfilled waste. As a result, the presence of MPs in both landfilled wastes and leachate was the primary issue with landfills, and researchers in this field are working to solve and monitor these pollutions. In addition, in average, 32% of wastes were landfilled in countries that had the MPs/PFAS ratio of more than one while in countries with ratio of lower than one, this amount was 20%.

By converting microplastics and polymers into carbon dioxide and mineral fractions, the incinerator can eliminate plastic waste.^46,47^ According to Fig. S5 in the ESI,† countries with higher incineration rates, such as Japan (71%), Norway (57%), and Denmark (54%), had lower MPs/PFAS ratios than countries with lower incineration rates, such as Australia (1%), Spain (10%), and Italy (21%). The average amount of incinerated waste was 27% in countries with a higher number of MPs publications (ratio of more than one), while 43% in countries with a higher number of PFAS publications (ratio of less than one). Although MPs may still be present in synthetic fibres derived from unburned materials during this process, but the concentration is negligible.^48^ In addition, the existence of MPs in bottom ash is highly related to the type of incinerated wastes and the method of incinerating. For example, the results of one study on effectiveness of incineration on microplastic removal revealed that the concentration of MPs in the bottom ash was negligible compared to landfill leachate, wastes and soils.^46,49^ As a result, it is possible to infer that incineration is an effective method of managing solid waste in terms of decreasing the concentration of MPs before releasing to the environment.

### Drinking water

Drinking water has been considered a dominant exposure pathway for PFAS and MPs over the last decade due to contamination of groundwater and surface water with these pollutants which raises public concern about human exposure risks to PFAS and MPs^50^ and strongly linked to a country's economic, social, and geographic conditions. Different parameters can cause these pollutions, which are classified into point and diffuse sources; for example, landfills and wastewater treatment plants are the primary point sources, while runoff is classified as a diffuse source.^50,51^

Table 7 shows the freshwater availability of G20 countries; based on the findings, the mean freshwater per capita for countries with a MPs/PFAS ratio of greater and lower than one was 6822 and 20 540 m^3^ per capita, respectively. The results also indicated that countries with higher freshwater availability per capita were more concerned about PFAS issues. Surface water and groundwater are both important sources of drinking water. For example, more than 70% of drinking water in Germany and Denmark comes from groundwater, while more than 80% of drinking water in Canada and the United States comes from surface water. Fig. S6 and S7 in the ESI† show the scatterplot of the numbers of publications in MPs and PFAS and the ratio of MPs/PFAS against the source of drinking water of G20 countries.

According to the results of Fig. S6 and S7 in the ESI,† the ratio of MPs/PFAS in countries that rely more on surface water as a source of drinking water was less than one, whereas in countries where groundwater is the primary source of drinking water, the ratio was greater than one. For example, more than 80% of drinking water in Canada, the United States, Japan, and China comes from surface water, with the MPs/PFAS ratios of 0.39, 0.41, 0.68, and 0.28, respectively. On the other hand, groundwater supplies more than 60% of drinking water in Germany, India, France, and the Netherlands, which had the MPs/PFAS ratios of 1.4, 1.2, 1.2, and 1.4, respectively.

### Wastewater

Sewage sludge in wastewater treatment plants (WWTPs) contains approximately 90% of the microplastics, cosmetic and hygiene product particles that have not been removed by conventional treatment procedures.^55–57^ In addition to MPs, the sludge from wastewater treatment plants also has PFAS, although PFAS-based products phased out in 2002.^58,59^ About one-third of the sludge from the WWTPs is used as fertilizer in agriculture which is considered a direct source, and the other half is landfilled, which both have negative impacts on groundwater as a slow and non-predictable source.^60–62^

According to Fig. S8 in the ESI,† the number of studies in countries with higher treated wastewater was lower than in countries with lower treated wastewater. In addition, as mentioned earlier, the sludge from conventional wastewater treatment contains a considerable amount of MPs; therefore, it can be concluded that by increasing the proportion of treated wastewater, the concerns about the concentration of MPs on the sludge have been increased; as a result, the ratio of MPs/PFAS in countries with higher proportion of treated wastewater was higher than other countries. For example, England, Germany, Netherlands, and Switzerland have the highest treated wastewater (more than 95%) with the highest MPs/PFAS ratio (more than 1.5). In addition, the mean proportion of treated wastewater in countries with a focus on MPs topics (MPs/PFAS ratio of more than one) was 90%, while the mean proportion of treated wastewater in countries with a ratio less than one was 85%.

Furthermore, according to a report on the wastewater treatment efficiency of different countries on the 2020 Environmental Performance Index (EPI), Yale University,^63^ countries with higher wastewater treatment scores focused more on MPs topics than PFAS topics, implying that the MPs/PFAS ratio was higher in countries with higher wastewater treatment rankings. For example, the ratios of MPs/PFAS in Germany, the Netherlands, Switzerland, Spain, and Australia, which have the highest scores (more than 99%) between G20 countries, were more than one.

### PCA and clustering of economic, social, and geographical factors for G20 countries

**Fig. 6:**
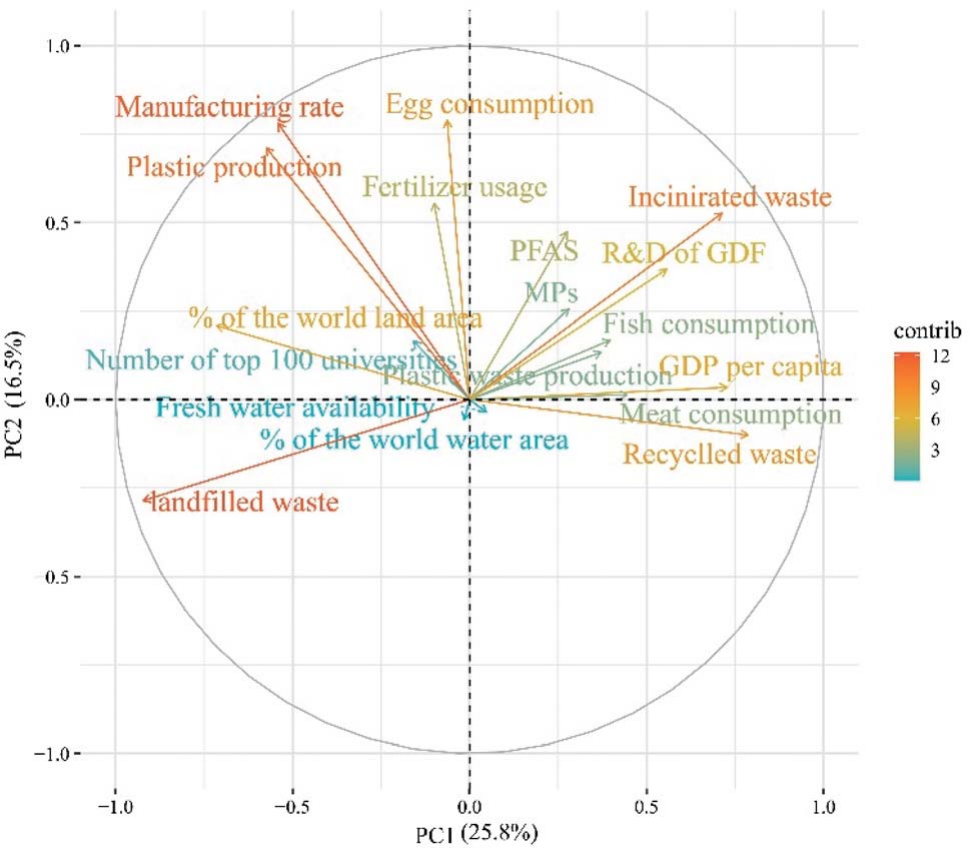
Two-dimensional principal component's plot of principal components 1 (PC1) and 2 (PC2) for the number of publications (PFAS and MPs; normalised with the total number of articles in each country) and all variables for G20 countries.

PCA provided valuable evidence of the most important variables between all analysed variables. A biplot chart of the first and second components can be seen in Fig. 6, with the higher explained variances, 25.8% (PC1) and 16.5% (PC2). Also, the larger the arrows and angle values near 0 or 180 degree between the variables and publication numbers (PFAS or MPs), respectively, indicate a more proportional or inversely proportional relationship. The angles between the publication numbers in PFAS and MPs and other variables are shown in Table 8. For example, incinerated wastes and R&D (as a percentage of GDP) have a proportional relationship with the number of PFAS publications, and in addition to these variables, plastic waste production and fish consumption have a proportional relationship with the number of MPs publications, since they all line up next to each other. While correlations between the number of publications on both topics and freshwater availability and landfilled wastes were found to be inversely proportional. Therefore, according to the results from Table 8, the number of publications in both records was more correlated with the variables in red, while the number of publications was inversely correlated with the variables in green.

**Table 8:** The angles between PCAs variables and the number of MPs and PFAS publications (scaled from green to red from higher angle to lower angles)

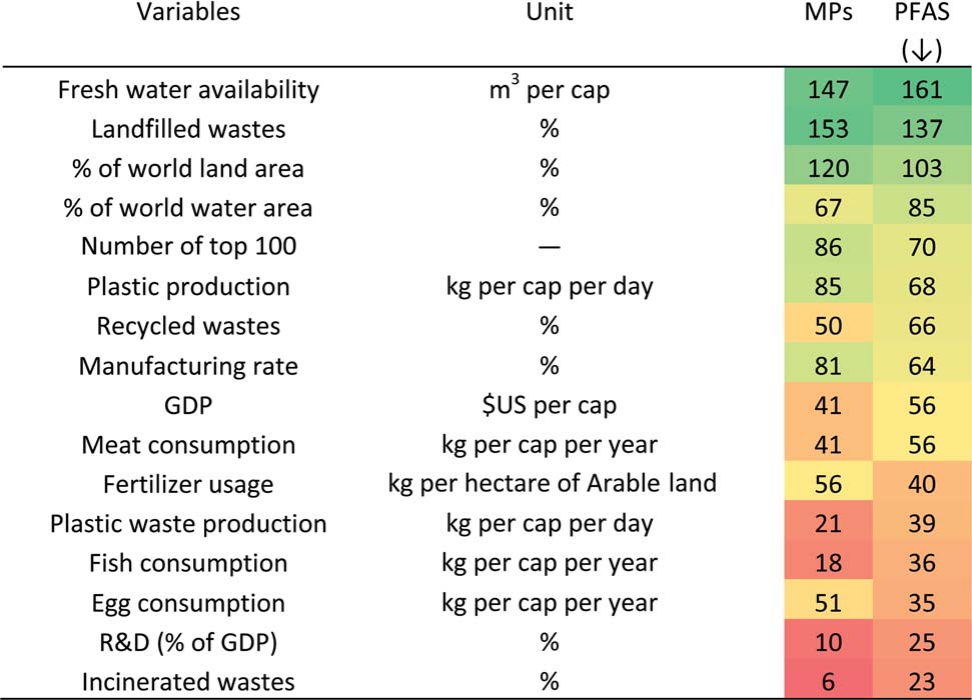

**Fig. 7:**
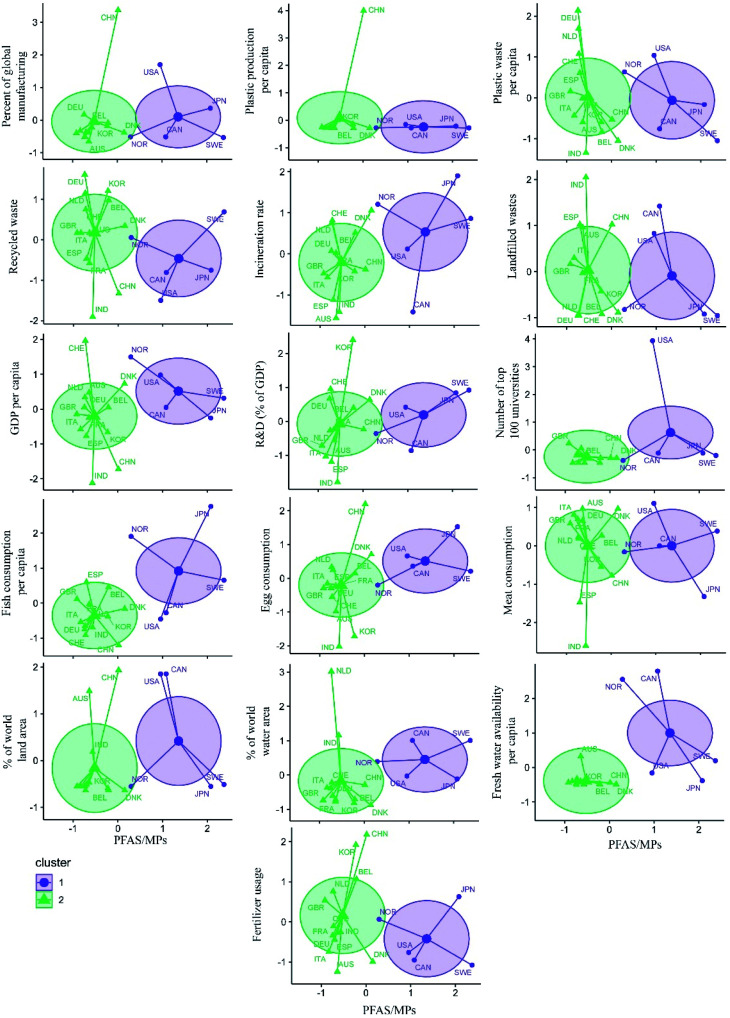
Scatter plot of PFAS/MPs ratio and different variables of G20 countries by cluster; AUS: Australia, BEL: Belgium, CAN: Canada, CHE: Switzerland, CHN: China, DEU: Germany, DNK: Denmark, ESP: Spain, FRA: France, GBR: England, IND: India, ITA: Italy, JPN: Japan, KOR: South Korea, NLD: Netherlands, NOR: Norway, RUS: Russia, SWE: Sweden, USA: United States of America.

Fig. 7 shows the clustering analysis of PFAS/MPs ratios with different variables listed in Fig. 1 for G20 countries. According to Fig. 7, countries in cluster 1, which had a higher percentage of global water area (with an average value of 6.5), focused on PFAS issues. The Netherlands and India, both of which were located outside of cluster 2, had an area greater than 6.5 percent but did not belong to cluster 1. As a result, it is possible to conclude that countries with more water are more concerned about PFAS issues.

In terms of fertiliser usage by countries, the average of this variable by countries in cluster 1 was 150 kilograms per hectare of Arable land, while the average of cluster 2 was 200 kilograms per hectare of Arable land. As a result, it can be concluded that countries with higher fertiliser usage focused on MPs issues.

In terms of freshwater availability, the average for countries in the cluster 1 was 36 000 m^3^ per capita, while the average for countries in cluster 2 was 3000 m^3^ per capita; thus, it can be concluded that the focus of countries with higher fresh water availability was on PFAS topics.

The same pattern can be seen for other variables, indicating that the focus of Sweden, Japan, Canada, the United States, and Norway was on PFAS issues. For example, the PFAS/MPs ratio was higher in cluster 1, which had a lower average of plastic production per capita than cluster 2, even without taking China into account, which has the highest plastic production among other countries. Furthermore, most countries in cluster 2 had higher manufacturing, recycling, and landfilling rates, as well as a lower percentage of global land and water area, GDP per capita, meat, egg, and fish consumption, and incineration rate.

### Microbeads

**Fig. 8:**
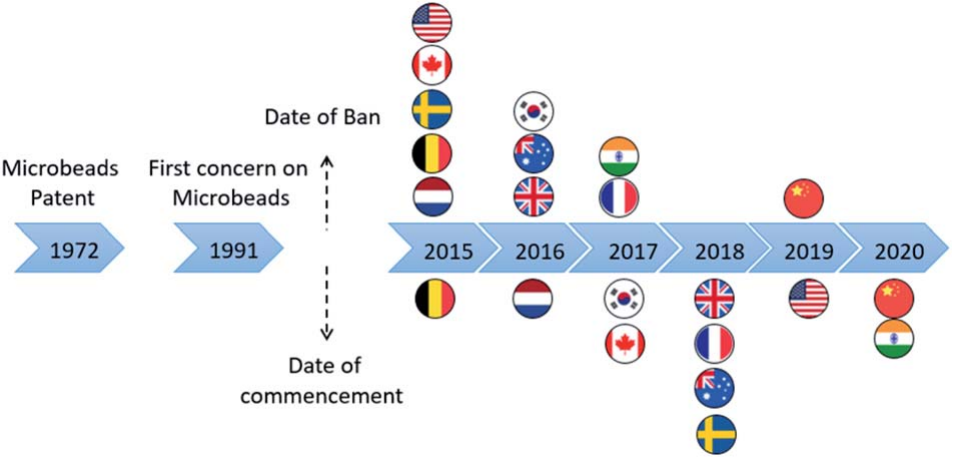
The timeline for proposed restrictions on microbeads.

As previously stated, microplastics can come from both direct and secondary sources; and microbeads have been identified as direct sources of microplastics, primarily used in cosmetics.^64^ Microbeads are also known as microspheres, plastic particulates, and ingredients in personal care and cosmetic products in the industry (PCCPs).^64^ Microbeads were patented in 1972 for use as exfoliating agents in cosmetics, and by the early 1990s, it was estimated that everyone used at least one microbead-containing scrub on a daily or weekly basis.^65^Fig. 8 shows the proposed timeline for microbeads restrictions.

**Fig. 9:**
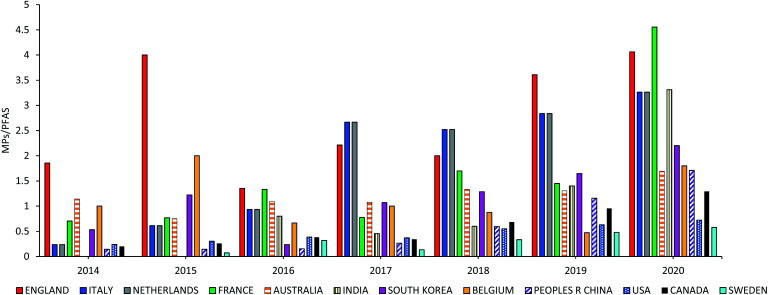
The ratio of MPs/PFAS for the countries from G20 with microbeads restrictions, which are shown in Fig. 8.

According to Fig. 9 and S9 in the ESI,† it can be concluded that the ratio of MPs/PFAS increased after the commencement of microbeads. In the Netherlands, for example, the MPs/PFAS ratio increased from 0.72 in 2016 to 4.6 in 2017 following the 2016 ban on microbeads. Furthermore, the ratio increased from 0.2 to 1.1 in South Korea, which banned microbeads in 2017.

## Conclusions

This article presented and discussed the scientific publication record from 1990 to 2020 on two topics: microplastics and perfluoroalkyl and polyfluoroalkyl substances. The goal is to visualise how the bibliometric and scientometric trends on the two topics (“PFAS” and “MPs”) have changed over time, using different classification perspectives such as country, year, source, and organisation, by extracting scientific publication records from Web of Science. The following are the key findings of this paper, which present the study's conclusions:

• It was discovered that in recent years (beginning in 2018), research topics related to MPs have surpassed PFAS topics, and the MPs/PFAS ratio has increased significantly from 0.2 in 2011 to more than 2.5 in 2020.

• PFAS research topics were more prevalent in countries with higher water areas compared to the land area, while for MPs, the trend was inverse.

• The number of MPs studies had proportional relationship with plastic waste production per capita, recycled and landfilled wastes.

• There was a direct relationship between the number of MPs publications and plastic waste production per capita.

• Strong positive correlation was discovered between the number of MPs publications and the amount of landfilled waste.

• The focus of countries with higher incineration rate was on PFAS topic like Japan and Norway.

• Countries with higher freshwater availability per capita were more concerned about PFAS issues.

• Countries that rely more on surface water as a source of drinking water was concerned more on PFAS topics, whereas in countries where groundwater is the primary source of drinking water, MPs were the dominant topics.

• The ratio of MPs/PFAS increased after the commencement of microbeads restrictions. In the Netherlands, for example, the MPs/PFAS ratio increased from 0.72 in 2016 to 4.6 in 2017 following the 2016 ban on microbeads.

## Author contributions

Reza Bakhshoodeh: performed the experiments; analysed and interpreted the data; contributed analysis tools; wrote the paper. Rafael M. Santos: conceived and designed the experiments; analysed and interpreted the data; wrote the paper.

## Conflicts of interest

There are no conflicts to declare.

## Supplementary Material

RA-012-D1RA09344D-s001
