# Supplementary material for: Comparative bibliometric trends of microplastics and perfluoroalkyl and polyfluoroalkyl substances: how these hot environmental remediation research topics developed over time
Source: RSC Adv. 2022 Feb 9;12(8):4973–87. doi: 10.1039/d1ra09344d (PMC8981264; doi:10.1039/d1ra09344d)
Supplement: RA-012-D1RA09344D-s001 [file RA-012-D1RA09344D-s001.pdf]

Electronic Supplementary Material (ESI) for RSC Advances.
This journal is © The Royal Society of Chemistry 2022

Supplementary Materials

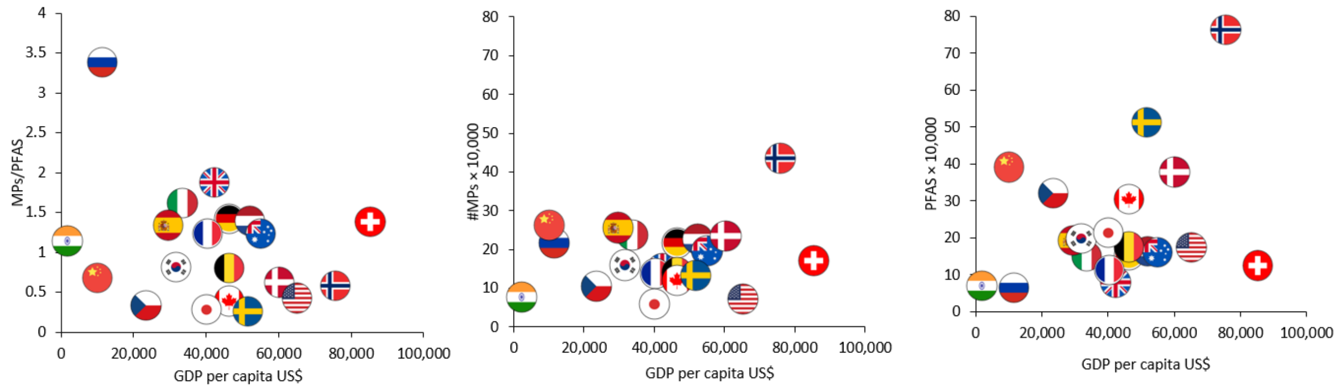

Fig. S.1. The number of publications in MPs, PFAS (normalised with the total number of articles in each country) and MPs/PFAS against the GDP per capita of G20 countries.

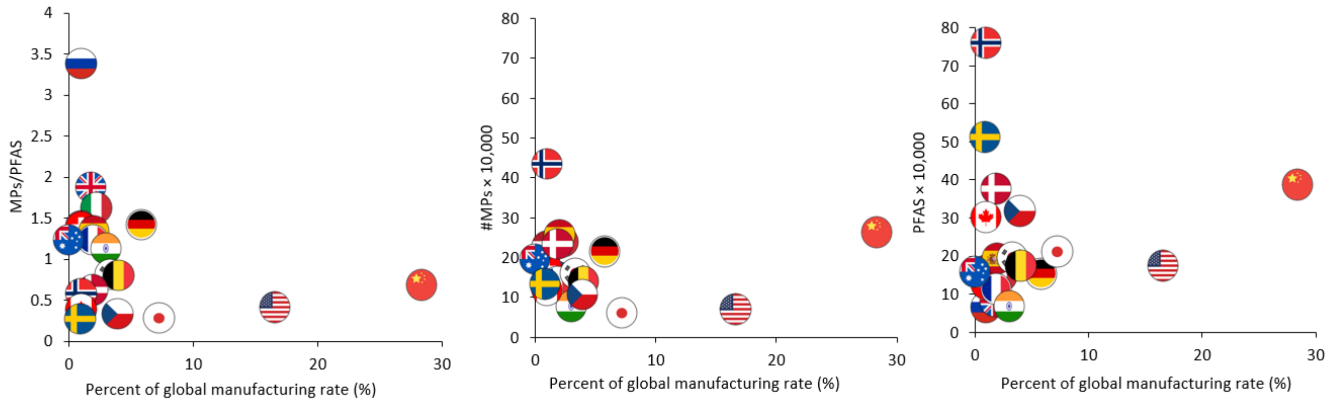

Fig. S.2. The number of publications in MPs, PFAS (normalised with the total number of articles in each country) and MPs/PFAS against the percent of global manufacturing (%) of G20 countries.

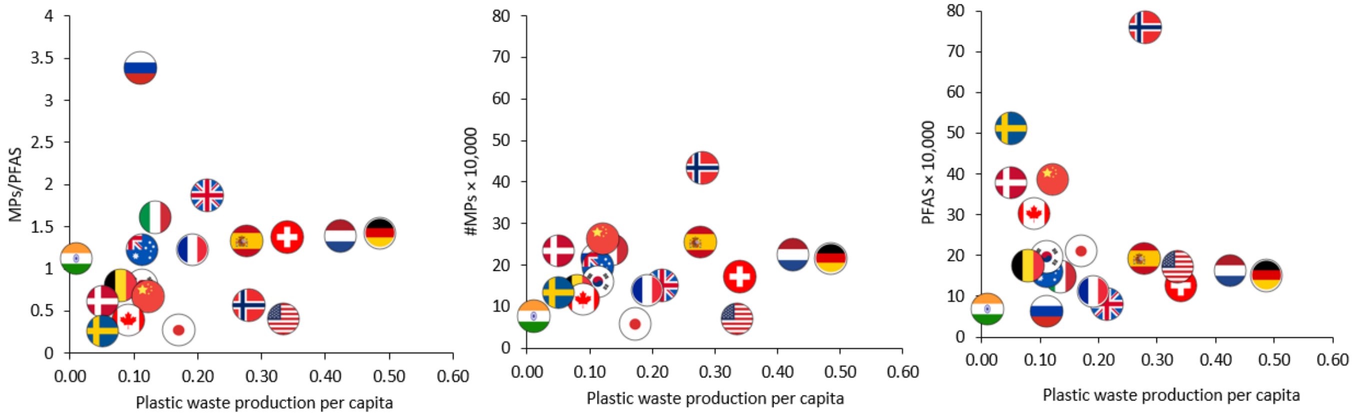

Fig. S.3. The number of publications in MPs, PFAS (normalised with the total number of articles in each country) and MPs/PFAS against the plastic wastes production per capita of G20 countries.

Supplementary Materials

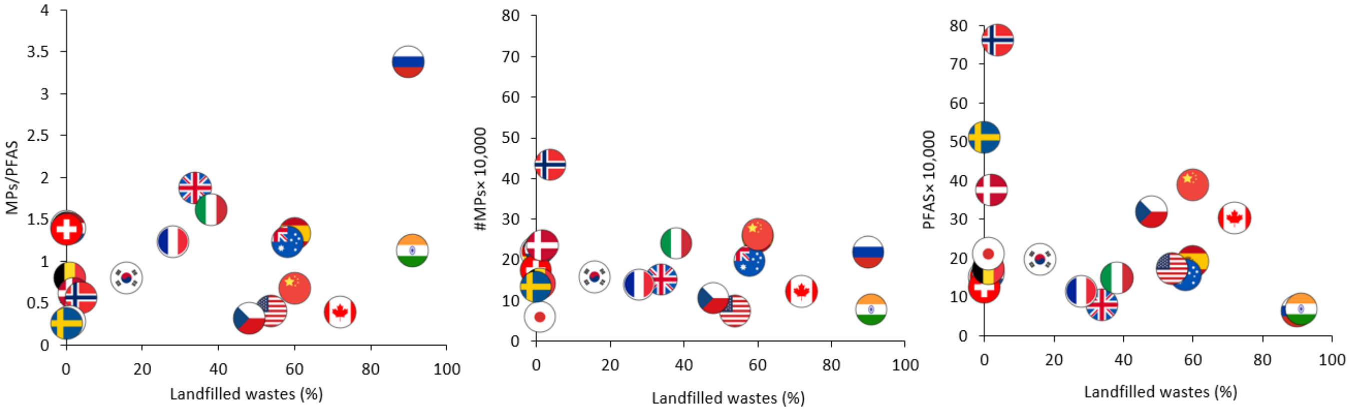

Fig. S.4. The scatterplot of the number of publications in MPs, PFAS (normalised with the total number of articles in each country) and MPs/PFAS against the ratio of wastes that have been landfilled of G20 countries.

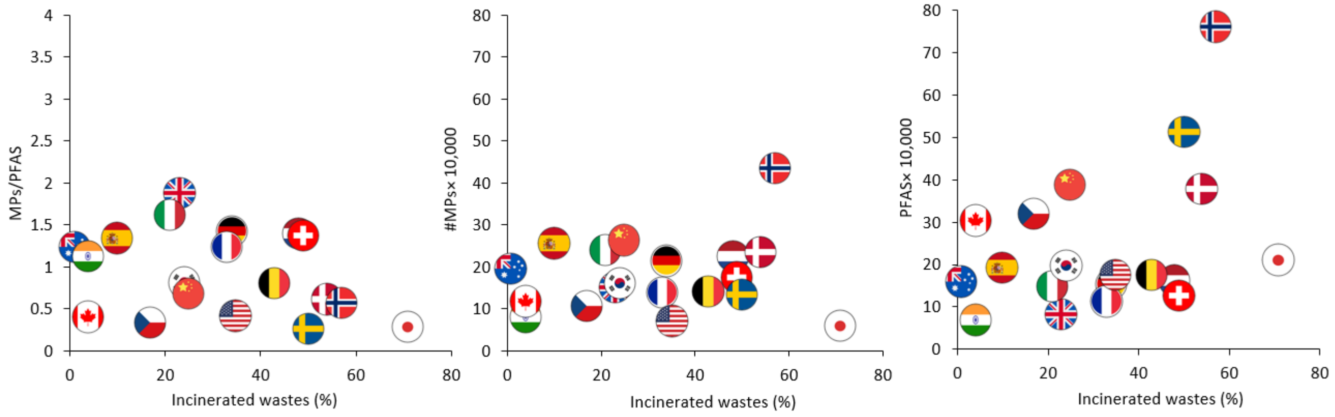

Fig. S.5. The scatterplot of the number of publications in MPs, PFAS (normalised with the total number of articles in each country) and MPs/PFAS against the ratio of wastes that have been incinerated of G20 countries.

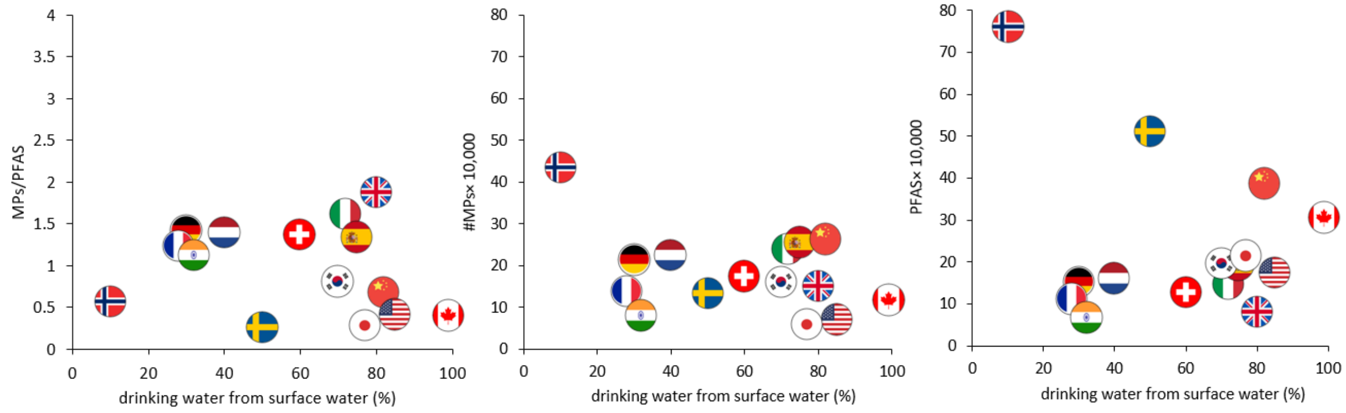

Fig. S.6. The scatterplot of the number of publications in MPs, PFAS (normalised with the total number of articles in each country), and MPs/PFAS against the ratio of surface-water point source as main drinking water source (% of the total pop.) of G20 countries.

Supplementary Materials

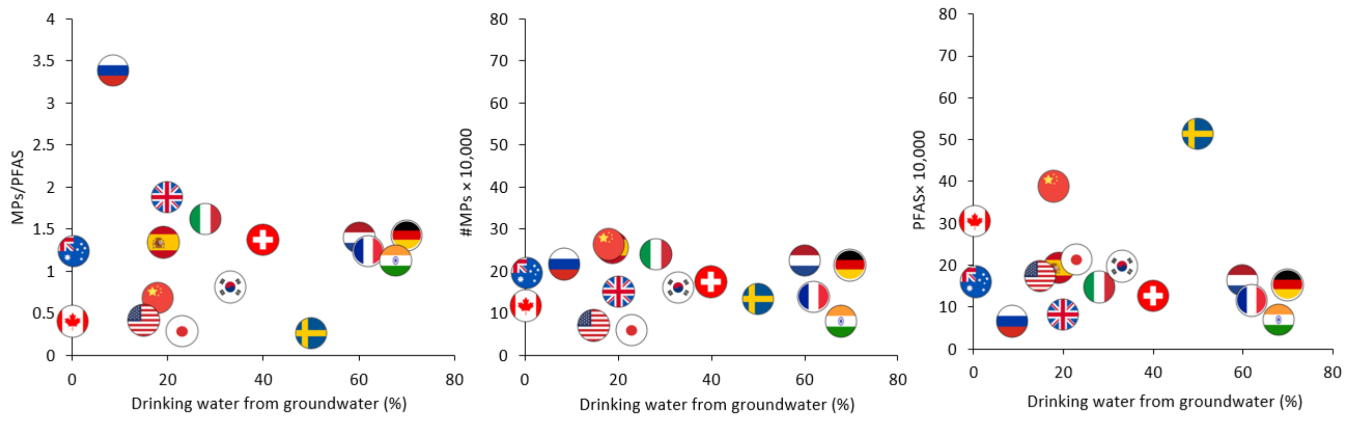

Fig. S.7. The scatterplot of the number of publications in MPs, PFAS (normalised with the total number of articles in each country), and MPs/PFAS against the ratio of groundwater point source as main drinking water source (% of the total pop.) of G20 countries.

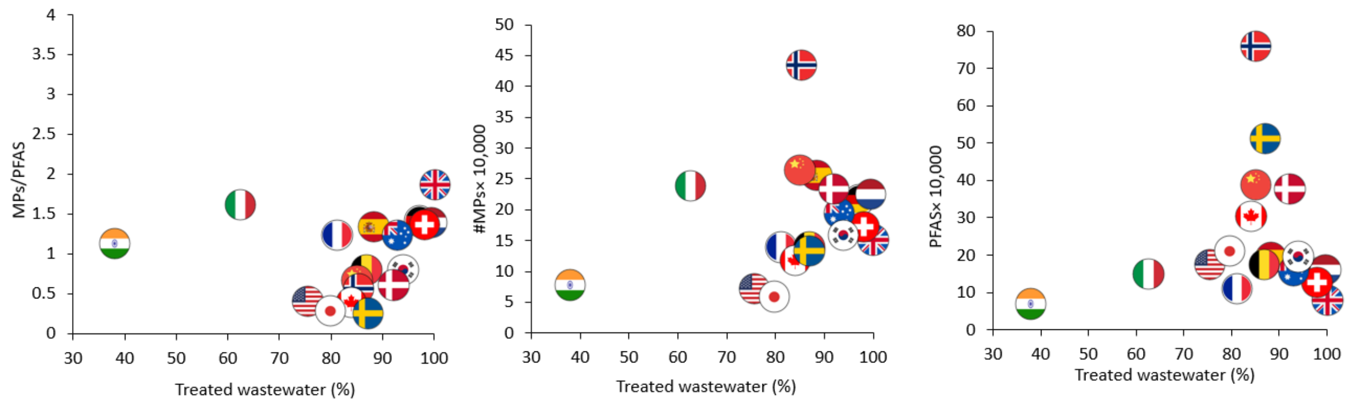

Fig. S.8. The scatterplot of the number of publications in MPs, PFAS (normalised with the total number of articles in each country), and the ratio of MPs/PFAS against the ratio of treated wastewater of G20 countries.

Supplementary Materials

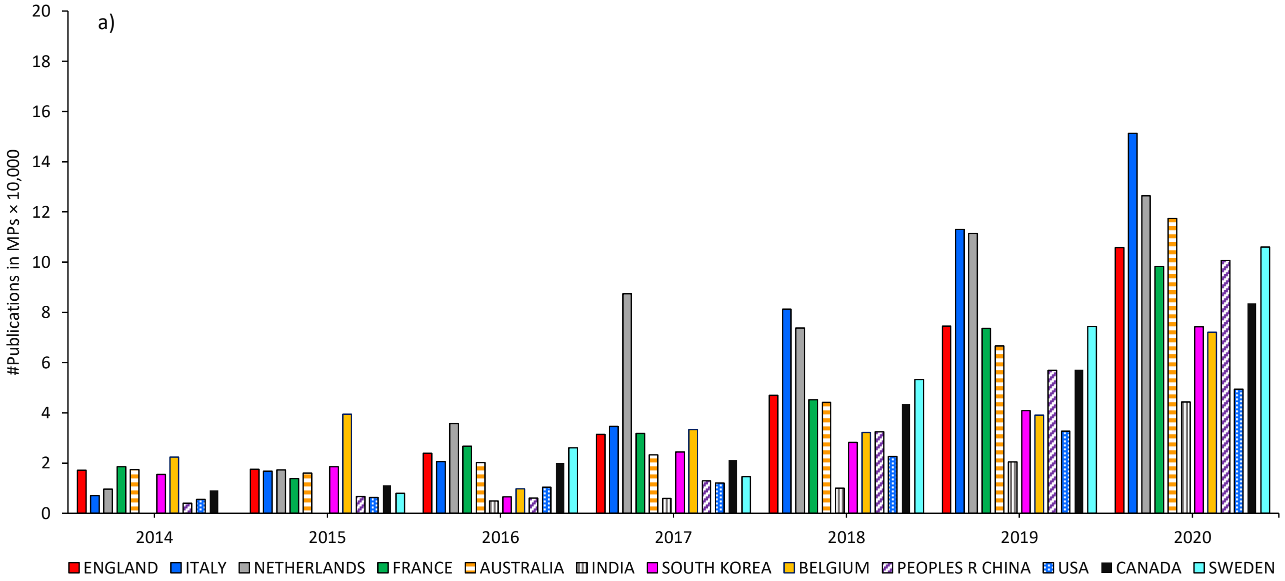

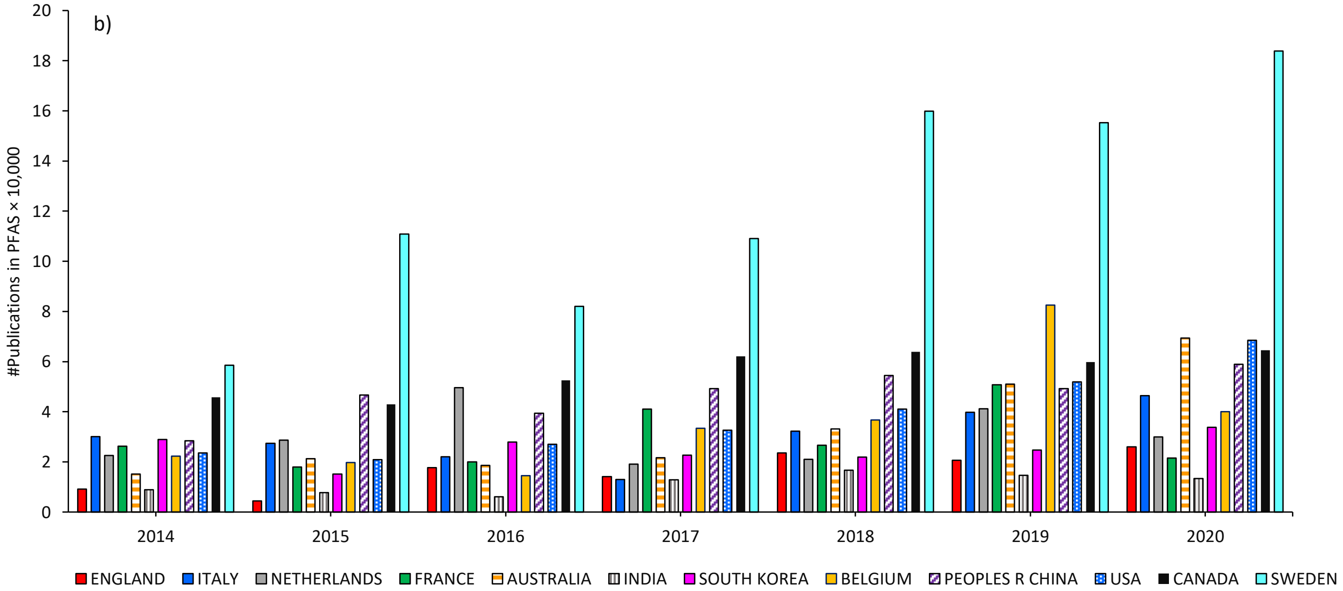

Fig. S.9. a) The number of publications (normalised with the total number of articles in each country) in MPs and b) PFAS for the countries from G20 with microbeads restrictions, which are shown in Fig. 8.
